# Buggy Sugar

**Published:** 1869-03

**Authors:** 


### BUGGY SUGAR.

The Editor believes he has eaten more sugar than any other two men of his size and age, and now as he is approaching a hundred years, finds himself as lively as a cricket or a newly made tadpole turned into a frog; and yet Mr. Robert Nicol, of Greenwich, Scotland, and Professor Cameron, of Dublin, have been taking a trial at microscopy, and say that they find in every teaspoonful of raw sugar about a thousand of the ugliest little wretches wriggling about, with horns and daggers ready to poke them through our vitals at any moment, and without the slightest compunction; in fact, they rather like it.

In plain phrase, there are about forty thousand of these living monsters in every pound of raw brown sugar. What a sight of them we must have devoured in our life-time; but, we think, it will be rather better to take to the use of refined sugar which is perfectly free from the insect.

Microscopic science seems to be revealing the fact, that every grain and fruit and vegetable has some living thing which revels, eats, lives and dies in it; tobacco, cotton, wheat, potatoes, all have their depredators and enemies. But science, while she reveals dangers we never dreamed of also finds a remedy, sooner or later; thorough cooking destroys the trichina of pork, and refined sugar has no "Acarus Sacchari."

